# Resistance to antihypertensive drugs targeting Renin-Angiotensin-Aldosterone-System in cancer patients: a case series

**DOI:** 10.1186/s40959-020-00071-x

**Published:** 2020-08-27

**Authors:** Mishita Goel, Rajiv Sunil Varandani, Tochukwu M. Okwuosa

**Affiliations:** 1 Department of Internal Medicine, WSU/Ascension Providence Rochester Hospital, 1101 W University Drive, Rochester Drive, Rochester, MI 48307 USA; 2 Chicago College of Osteopathic Medicine, Midwestern University, Chicago, IL USA; 3 Department of Internal Medicine, Rush University Medical Center, Chicago, IL USA

**Keywords:** Hypertension, Angiotensin-converting enzyme inhibitors, Angiotensin receptor blockers, Chemotherapeutic agents

## Abstract

Hypertension impacts overall prognosis in cancer patients. There are no specific recommendations for its management in these patients. We report a case series of 5 cancer patients with suboptimal BP lowering and even worsening BP with ACEi or ARBs that improved to normal upon discontinuation of these drugs.

## Learning points

- Hypertension is one of the most common comorbidities significantly impacting prognosis in cancer patients but still there are no specific recommendations for its management in these patients.
- Antihypertensive drugs targeting the Renin Angiotensin Aldosterone System (RAAS) are commonly used for management of hypertension in cancer patients. Preclinical studies in rats have demonstrated suboptimal blood pressure lowering effects of these agents in severe hypertension, however clinical experience with use of these agents for management of hypertension in cancer patients has never been reported.
- Our study involved 5 cancer patients with uncontrolled hypertension managed with Angiotensin-converting enzyme inhibitors (ACEi) and Angiotensin Receptor Blockers (ARBs) that later improved to normal upon discontinuation of these drugs.
- There are very few current studies that have reported beneficial outcomes in patients on anti-angiogenic based cancer therapy with specific anti-hypertensive medication classes. Clinical experience with the use of specific anti-hypertensive medication classes in patients with cancer needs to be reported in a disciplined fashion.

## Introduction

Hypertension is one of the most common comorbidities reported in cancer patients. Chemotherapeutic agents, especially VEGF signaling pathway (VSP) inhibitors can not only worsen but cause de novo hypertension [1]. There are no specific recommendations for management of hypertension in cancer patients despite its significant impact on prognosis compared to any of the other cardiovascular risk factors in these patients [2]. Angiotensin-converting enzyme inhibitors (ACEi) are most commonly used to manage hypertension. Dirix et al. [3] reported continued increase in blood pressure (BP) despite addition of an ACEi in a 51-year old male with renal cell carcinoma until treated successfully with a long acting nitrate. Thus their efficacy in reducing BP in cancer patients needs further exploration. Herein we present a case series of five cancer patients with uncontrolled hypertension while being managed with ACEi or Angiotensin Receptor Blockers (ARBs), which became controlled after discontinuation of these drugs. Baseline characteristics of these patients are indicated in Table 1**.**

**Table 1:** Baseline Characteristics of the Series Patients

Case	Age	Gender	Ethnicity	Cancer Type	Chemotherapy/ Radiotherapy	Initial BP Range^a^	Final BP Range^b^
1.	77	Female	African- American	Multiple Myeloma	Bortezomib	180–190/ 70–80	125–135/ 60–65
2.	85	Male	Caucasian	Prostate Cancer with multiple bone metastasis	Androgen deprivation therapy, External beam radiation therapy	140–150/ 70–80	125–135/ 70–80
3.	79	Female	Caucasian	Ovarian carcino- sarcoma	Bevacizumab	160–180/ 70–80	120–130/ 60–80
4.	65	Female	Caucasian	Ovarian cancer with metastasis	Bevacizumab and cyclophosphamide	160–170/ 90–100	120–140/ 80–90
5.	72	Female	Caucasian	Breast cancer	Trastuzumab	190–210/ 80–90	110–120/ 56–74

^a^ Range of home blood pressure readings/log at presentation

^b^ Final BP means range of home blood pressure readings/log when taken off ACEi and/or ARB

*Abbreviations*: *ACEi* Ace inhibitor, *ARB* Angiotensin receptor blocker

We reviewed the charts of 5 adult cancer patients with resistant hypertension on ACEi and/or ARBs presenting to our cardio-oncology clinic over a 2-year period. The mean of 3 clinic or 2-week ambulatory blood pressure readings before starting each BP medication, after starting it, following any dose change, and after discontinuation of these drugs were recorded. All 5 patients were adherent with their medications, had laboratory workup for secondary hypertension in our cardio-oncology clinic, and had normal renal artery duplex scans.

## Case 1

**Fig. 1:**
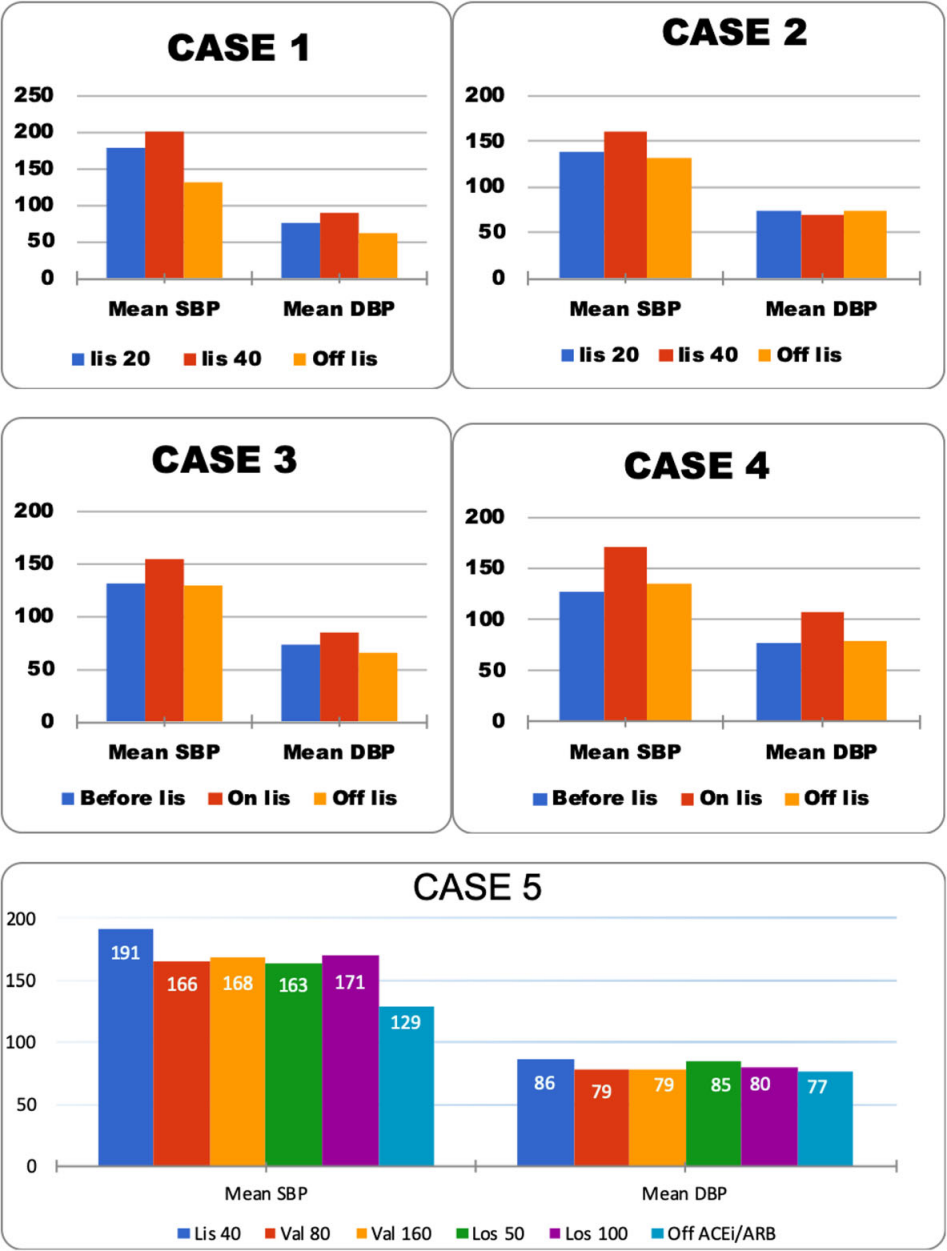
Comparison of average SBP and DBP readings in: Case 1. 77 year-old female with multiple myeloma on chemotherapy with bortezomib. Graph shows SBP and DBP on low, high dose, and off ACEi therapy. Case 2. 85 year-old male on ADT with leuprolide and EBRT for prostate cancer. Graph shows SBP and DBP on low, high dose, and off ACEi therapy. Case 3. 79 year-old female on bevacizumab immunotherapy for ovarian carcinosarcoma. Data shows SBP and DBP before and after starting ACEi therapy and after discontinuing it. Case 4. 65 year-old female on chemotherapy with bevacizumab for ovarian cancer. Data shows SBP and DBP DBP before and after starting ACEi therapy and after discontinuing it. Case 5. 72 year-old female on cancer therapy with trastuzumab for breast cancer. Data shows SBP and DBP on and later off ACEi and ARB therapies

A 77-year-old African-American female with history of IgG-Kappa multiple myeloma, aplastic anemia with Idiopathic Thrombocytopenic Purpura (requiring multiple transfusions) and type 2 diabetes mellitus (DM), presented for management of uncontrolled hypertension while being treated with bortezomib chemotherapy. Her BP log revealed systolic and diastolic blood pressure (SBP and DBP) ranges of 180-190 mmHg and 70-80 mmHg respectively, on an antihypertensive regimen including lisinopril 20 mg daily, felodipine 10 mg daily and spironolactone 25 mg daily. Her BPs remained elevated and increased even further on higher dose lisinopril of 40 mg daily. Lab data revealed elevated creatinine. After extensive workup, lisinopril was discontinued and carvedilol 12.5 mg and hydralazine 25 mg twice daily were added to her BP regimen. Her creatinine levels subsequently improved with improved BP readings ranging from 125 to 135/60-65 mmHg (Fig. 1).

## Case 2

An 85-year-old Caucasian male with multiple cardiovascular comorbidities (coronary artery disease, heart failure with preserved ejection fraction (HFpEF), DM, abdominal aortic aneurysm and brain aneurysm), Chronic Obstructive Pulmonary Disease and Aplastic anemia, was treated with External Beam Radiation Therapy (EBRT) and adjuvant androgen deprivation therapy (ADT) with Leuprolide for prostate cancer with multiple bone metastases. He presented to our cardio-oncology clinic for management of uncontrolled hypertension with SBP ranges of 140-150 mmHg. His anti-hypertensive regimen included spironolactone 25 mg daily, amlodipine 5 mg daily and lisinopril 20 mg daily. Amlodipine was discontinued due to significant lower extremity edema, and lisinopril was increased to 40 mg daily. Subsequent 2-week BP log revealed increased SBP readings to a range of 150-160 mmHg. Lisinopril was then discontinued and hydralazine was added to his BP regimen. At a dose of 50 mg QID, his BP readings normalized to 125–135/70-80 mmHg (Fig. 1).

## Case 3

A 79-year-old Caucasian female with BRCA2 gene mutation and Stage IIIC ovarian carcinosarcoma was referred for management of uncontrolled hypertension while on bevacizumab targeted therapy. Her BP was well controlled with spironolactone 25 mg daily and metoprolol succinate 25 mg BID. However due to concerns for bradycardia (HR 40–60 per min), metoprolol was discontinued. Following this, her SBP readings were slightly elevated in range of 140-150 mmHg, so lisinopril 10 mg daily was initiated. The patient’s SBP remained elevated and in fact worsened, ranging from 160 to 180 mmHg despite an increase in lisinopril dose to 20 mg daily. Lisinopril was then discontinued, and her BP readings normalized to 120–130/60-80 mmHg after re-initiation of low dose metoprolol succinate 25 mg BID (Fig. 1).

## Case 4

A 65-year-old Caucasian female with history of stage IV ovarian cancer presented with uncontrolled BP since she began treatment with bevacizumab and cyclophosphamide. Her hypertension had been well managed on carvedilol 12.5 mg BID prior to bevacizumab therapy, but she now had elevated BPs in the range of 160 s/90s on this drug. Lisinopril 20 mg daily was initiated for control of her bevacizumab-induced hypertension; but her BPs increased further, ranging from 170 to 180/85-115 mmHg. Lisinopril was subsequently discontinued, and she was initiated on BP therapy with amlodipine 5 mg daily. Following this change, her home BP readings significantly improved, with subsequent ranges of 120–140/80-90 mmHg (Fig. 1).

## Case 5

A 72-year-old Caucasian female with history of breast cancer s/p surgery and intraoperative radiation therapy, DM, and dyslipidemia, presented for management of uncontrolled hypertension while on cancer therapy with trastuzumab. Her SBP and DBP readings were found to be between 190 and 210 mmHg and 80–90 mmHg respectively on metoprolol 100 mg daily, lisinopril 40 mg daily, hydralazine 75 mg TID, and chlorthalidone 25 mg daily. Her antihypertensive regimen was adjusted to carvedilol 25 mg BID, hydrochlorothiazide 25 mg daily, amlodipine 10 mg daily and losartan 50 mg daily due to chronic cough on lisinopril. Losartan was later increased to 100 mg daily but BP remained elevated, and even worsened on this regimen (Fig. 1). Losartan was finally switched to nifedipine 120 mg daily; after which her BP readings declined to 120–140/70-80 mmHg.

## Discussion

Hypertension is the most common comorbidity seen in cancer patients. In fact many studies have shown it to be a risk factor for cancer due to abnormal proliferative pathways [4]. Cancer therapeutic agents such as bevacizumab, sunitinib, sorafenib, etc. are known to increase BP by decreasing endothelial nitric oxide (NO) production due to VEGF inhibition [5]. Trastuzumab, which is a humanized monoclonal antibody against HER2, also decreases VEGF expression [6]. EBRT causes a decrease in bioavailability of NO by impairing endothelium-dependent vasodilation of conduit arteries [7]. Other cancer therapies such as leuprolide can also cause hypertension resulting in increased cardiovascular events [8].

We observed resistance to antihypertensive agents targeting RAAS (ACEi/ARB) in our cancer patients; a phenomenon also observed in African American patients treated with ACEi/ARB monotherapy for hypertension. All of our patients had a history of hypertension prior to being initiated on chemotherapy. Their BPs were uncontrolled on ACEi/ARB but normalized on discontinuation of these agents. All of these patients reported adherence with their medications and had normal renal artery duplex scans. None of the physical exam findings or laboratory values suggested other causes of secondary hypertension.

Three of our patients were on bevacizumab and trastuzumab, which are known to decrease VEGF expression. Preclinical experiments in rats have shown an inability of ACEi to modulate higher increases in BP induced by VEGF inhibition, and suggest effectiveness in treatment for only mild increases in BP (10–15 mmHg) [9]. They also observed reduced renin levels in the rats exposed to higher levels of cediranib (a potent VSP inhibitor), and thus concluded that RAAS gets downregulated to maintain normotension when exposed to these agents. Other preclinical studies have also shown suppression of RAAS by angiogenesis inhibition [5]. Thus, the significance of RAAS in mediating antiangiogenic therapy-induced hypertension is still controversial and other mechanisms such as inhibition of endothelial derived relaxation factors, capillary rarefaction and alteration in pressure-natriuresis relationship, as well as other vasoconstrictive pathways, play a major role [10]. Thus ACEi/ARBs can cause suboptimal BP lowering effects in cases of severe hypertension due to already suppressed RAAS in these patients. Nonetheless, in vivo studies have shown that ACEi increases release of NO and are thus recommended as first line agents for management of anti-VEGF induced hypertension [11]; especially for their renoprotective effects given higher risk of proteinuria on VSP inhibition therapy [12]. It is possible that BP control with ACEi/ARBs in this population occurs due to angiogenesis inhibition by RAAS antagonism and not due to their direct antihypertensive action.

Recommendations for agents best used in the management of hypertension in patients on cancer therapy are variable and somewhat controversial, particularly for those on VEGF inhibitors. ACEi/ARBs are commonly preferred in cancer patients due to improved mortality outcome [13, 14]. However, as discussed, these agents could lead to suboptimal BP lowering as a result of RAAS suppression; particularly in cases of severe VEGF inhibitor-induced hypertension. This mechanism is similar to that of the repressed RAAS system leading to ACEi/ARB resistant hypertension in persons of African ancestry [15], and is a possible explanation for the uncontrolled hypertension observed in our case 1. This patient was also on treatment with bortezomib – a proteasome inhibitor that can rarely cause endothelial dysfunction leading to hypertension and vascular dysfunction [16]. Case 2 was on cancer treatment with leuprolide ADT which is known to cause hypertension. However, the resistance to ACEi observed in Case 2 was contrary to expectation since both leuprolide and EBRT are implicated in causing hypertension by mechanisms related to impairment of endothelium-dependent vasodilation, thus, ACEi/ARBs were expected to be effective [7, 8]. All these patients showed resistance to ACEi/ARB on different cancer therapies with malignancy being the only commonality. Indeed, patients with various cancers are known to overexpress Angiotensin II receptor 1, which is involved in BP regulation [17].

Our clinical experience shows that for those patients resistant to ACEi/ARBs, peripheral arterial vasodilators like hydralazine and dihydropyridine calcium channel blockers (CCBs) like amlodipine or nifedipine are more effective in managing hypertension in these patients. Curwen et al demonstrated reversal of marked captopril-resistant hypertension induced by cediranib in rats after treatment with nifedipine [10]. Clinical studies have also shown effective BP management with dihydropyridine CCBs after treatment with bevacizumab [18, 19]. In a recent study by Wiliany et al. including patients with mRCC, ACEi/ARBs were not associated with decreased BP during anti-VEGF TKI treatment while CCBs and potassium sparing diuretics were associated with significant reduction in BP [20]. Furthermore, long acting nitrates that increase NO bioavailability have also been shown to effectively control hypertension in patients on antiangiogenic therapy that was refractory to ACEi and CCBs [3]. However, there is a potential risk of compromising antiangiogenic benefits as preclinical evidence suggest the role of endothelial NO production in VEGF associated angiogenesis [21, 22].

In conclusion, possible mechanisms of hypertension resistant to ACEi/ARB observed in certain cancer patients include overexpression of Angiotensin II receptor 1, low renin state or RAAS suppression. Thus, the efficacy of drugs targeting RAAS for BP control in this population on active VEGF-inhibitor or other cancer therapy is still unclear. Since there are no specific recommendations for management of hypertension in cancer patients, clinical experience with its management needs to be reported. The observation of difficult-to-treat hypertension with ACEi/ARBs in cancer patients on VSP inhibitor or other therapy requires further investigation.

## Data Availability

Data sharing is not applicable to this article as no datasets were generated or analyzed during the current study.

## Abbreviations

ACEi: Angiotensin-Converting Enzyme inhibitors
ARBs: Angiotensin Receptor Blockers
VSP: Vascular Signaling Pathway
HER2: Human Epidermal Growth Factor receptor 2
RCC: Renal Cell Carcinoma
DM: Diabetes Mellitus
BP: Blood Pressure
SBP: Systolic Blood Pressure
DBP: Diastolic Blood Pressure
QID: 4 times daily
HR: Heart rate
PAI: Plasminogen activator inhibitor
NO: Nitric Oxide
RAAS: Renin Angiotensin Aldosterone System
VEGF: Vascular Endothelial Growth Factor
CCBs: Calcium channel blockers
